# Maternal Secretor Status Affects Oral Rotavirus Vaccine Response in Breastfed Infants in Bangladesh

**DOI:** 10.1093/infdis/jiaa101

**Published:** 2020-03-11

**Authors:** Frank B Williams, Abdul Kader, E Ross Colgate, Dorothy M Dickson, Marya Carmolli, Muhammad Ikhtear Uddin, Salma Sharmin, Shahidul Islam, Taufiqur Rahman Bhuiyan, Masud Alam, Uma Nayak, Josyf C Mychaleckyj, William A Petri, Rashidul Haque, Firdausi Qadri, Beth D Kirkpatrick, Benjamin Lee

**Affiliations:** 1 The University of Vermont Vaccine Testing Center, Larner College of Medicine, University of Vermont, Burlington, Vermont, USA; 2 International Centre for Diarrhoeal Disease Research, Bangladesh, Dhaka, Bangladesh; 3 Department of Public Health Sciences and Center for Public Health Genomics, University of Virginia, Charlottesville, Virginia; 4 Division of Infectious Diseases and International Health, University of Virginia, Charlottesville, Virginia

**Keywords:** Rotavirus, Oral vaccine, Secretor, Breast Milk

## Abstract

Secretor status controls mucosal histo–blood group antigen expression and is associated with susceptibility to rotavirus (RV) diarrhea, with nonsecretors less susceptible to symptomatic infection. The role of breast milk secretor status on oral live-attenuated RV vaccine response in breastfed infants has not been explored. In a monovalent G1P[8] RV vaccine (Rotarix) trial in Bangladesh, RV-specific plasma immunoglobulin A antibody seroconversion rates were higher among infants of maternal nonsecretors (39%) than infants of maternal secretors (23%; *P *= .001). Maternal status remained a significant predictor when correcting for infant status (*P *= .002). Maternal secretor status should be considered when interpreting oral RV vaccine responses in low- and middle-income settings.

***Clinical Trials Registration.*** NCT01375647.

Rotavirus (RV) infection is a leading cause of diarrheal disease and death in children, accounting for approximately 215 000 pediatric deaths annually [1]. Most deaths occur in low- and middle-income countries [1], where oral vaccines demonstrate lower immunogenicity and efficacy than in high-income countries. The biologic basis for vaccine underperformance is an area of intense research interest. Proposed explanations include variations in host histo–blood group antigen (HBGA) expression and breast milk composition [2].

HBGAs are oligosaccharides (glycans) that can be expressed on mucosal surfaces and bind human RVs [3]. These glycans are also expressed in breast milk as human milk oligosaccharides (HMOs). The activity of α[1,2]-fucosyltransferase, encoded by the *FUT2* gene, determines “secretor” versus “nonsecretor” status and HBGA expression. Secretors can express 2-fucosylated oligosaccharides in breast milk and H-type 1 antigens on mucosal surfaces. Homozygotes for inactivating alleles in *FUT2* are characterized as nonsecretors and lack this ability but seem to be protected against symptomatic RV infections [4, 5].

Both immunoglobulin and nonantibody milk components have been implicated in decreased RV vaccine infectivity in vitro, but most clinical trials have found no benefit from withholding breastfeeding at the time of vaccination [2]. However, these trials have not accounted for maternal secretor status. Therefore, we explored the effect of breast milk secretor status on oral RV vaccine immunogenicity among infants in Bangladesh.

## METHODS

We performed a substudy within Performance of Rotavirus and Oral Polio Vaccines in Developing Countries (PROVIDE), a birth cohort study that included an open-label Rotarix (GlaxoSmithKline) vaccine efficacy (VE) trial conducted in Dhaka, Bangladesh from 2010 to 2014 [6]. PROVIDE was approved by the ethical review boards of the International Centre for Diarrhoeal Disease Research, Bangladesh, the University of Vermont, and the University of Virginia. The study was registered at ClinicalTrials.gov (NCT01375647). All families participating provided signed informed consent. Infants randomized to receive Rotarix received the vaccine at 10 and 17 weeks. Infant blood specimens were collected at weeks 6 and 18 for plasma RV-specific immunoglobulin (Ig)A (RV-IgA) and RV-specific IgG (RV-IgG) measurement with enzyme immunoassay (EIA) [6]. Infant saliva was collected at 1 or 2 years of age. Maternal peripheral blood mononuclear cells (PBMCs) and breast milk samples were collected at a single time point at or before week 6.

Infant secretor phenotype was determined in saliva using Lewis antigen dot-blot assays and *Ulex europaeus* Agglutinin 1 (UEA-1) EIAs [4]. Seroconversion was defined as postvaccination RV-IgA level ≥20 U/mL at 18 weeks after week 6 prevaccination concentration <20 U/mL. To determine breast milk phenotype, Lewis antigen EIA was performed on milk samples using antibodies specific for Lewis a and b (Supplementary Methods) [7]. For all specimens, secretor status was inferred by Lewis antigen: Lewis^a+b−^ was classified as nonsecretor, and Lewis^a−b+^ and Lewis^a+b+^ as secretor. The UEA-1 EIA could not be validated on breast milk, thus Lewis^a−b−^ mothers were categorized as indeterminate and excluded from secretor analysis. Peak RV season was defined as months demonstrating a >50% higher incidence of RV diarrhea (RVD) compared with the mean annual incidence in the PROVIDE cohort. Weeks of peak season exposure were quantified as number of weeks an infant was exposed to peak RV season between 6 and 18 weeks of age.

To confirm observed phenotype associations, maternal *FUT2* genotyping was performed in mothers with available PBMCs. Full details are provided in the Supplementary Methods. Briefly, DNA was extracted from PBMCs for polymerase chain reaction and Sanger sequencing of the coding region (exon 2) of *FUT2*, using primers described elsewhere [8, 9]. Homozygotes for nonsecretor alleles, including rs601338G>A, the most prevalent nonsecretor single-nucleotide polymorphism that encodes a stop codon, and rs200157007C>T, a missense mutation that is common in Bangladeshi populations, were classified as *se/se*; homozygotes and heterozygotes for functional alleles were classified *Se/Se* and *se/Se,* respectively.

The primary outcome was infant RV-IgA seroconversion. Categorical outcomes were assessed using χ ^2^ or Fisher exact test to estimate proportion differences with corresponding 95% confidence intervals (CIs) and associated relative risks (RRs). Simple and multiple logistic regression were used to analyze infant and maternal contributions to seroconversion. Analyses were performed using IBM SPSS software, version 25 (IBM). Differences were considered statistically significant at *P* < .05 (2 sided).

## RESULTS

Of 350 vaccinated infants, seroconversion and secretor phenotype data were available for 274 mother-infant pairs. Twenty-eight Lewis-negative mothers were excluded from secretor analysis, leaving 246 mother-infant dyads. Seventy-four mothers (30%) and 71 infants (28%) were nonsecretor by phenotype. Maternal secretor status was not associated with breast milk RV-IgA or infant prevaccination RV-IgG concentration (maternal antibodies; Table 1). Seroconversion was more frequent among infants born to nonsecretor mothers than among infants born to secretor-positive mothers (39% vs 23%; RR, 1.69; 95% CI, 1.14–2.50) (Table 2). Infant secretor status was not significantly associated with seroconversion: 18 (25%) of the nonsecretor infants seroconverted, compared with 51 (29%) of secretor-positive infants (RR, 0.82; 95% CI, .53–1.27). The effect of maternal status was more apparent in secretor-positive infants (nonsecretor vs secretor-positive mothers, 55% vs 24%; RR, 2.32; 95% CI, 1.50–3.59) than in nonsecretor infants (28% vs 22%, respectively; RR, 1.30; 95% CI, .55–3.07). When seroconversion was defined as a 3-fold rise in RV-IgA, the rate of seroconversion remained significantly higher for infants of nonsecretors (38% vs 24%; *P *= .03).

**Table 1.** Characteristics by Maternal Secretor Phenotype^a^

	Mother-Infant Pairs, No. (%)		
Characteristic	Total (N = 246)	Maternal Secretors (n = 172)	Maternal Nonsecretors (n = 74)
Maternal, infant, household and socioeconomic features			
Infant sex, female	130 (53)	95 (56)	35 (47)
Maternal age at enrollment, y	25 (22−28)	25 (22–28)	25 (22–28)
Cesarean mode of delivery	48 (20)	32 (19)	16 (22)
Total monthly income, median, takas	10 000 (3000–77 000)	10 000 (3000–77 000)	10 000 (3000–77 000)
Any maternal education	172 (70)	115 (70)	50 (68)
Access to water treatment	99 (40)	72 (42)	27 (37)
Continued breastfeeding through 18 wk	244 (99)	170 (99)	74 (100)
Infant Lewis negative status	10 (4.1)	8 (4.7)	2 (2.7)
Immunogenicity			
GMT (95% CI), U/mL			
Infant serum RV-IgA at wk 6	9 (8–9)	9 (8–10)	8 (8-9)
Infant serum RV-IgG at wk 6^b^	15 180 (11 902–19 331)	16 044 (11 917–21 611)	13 364 (8512–21 935)
Breast milk RV-IgA^c^	2735 (2048–3621)	2638 (2004–3486)	2894 (1578–5317)
Infant serum RV-IgA seropositivity at wk 6	16 (7)	12 (7)	4 (5)

Abbreviations: CI, confidence interval; GMT, geometric mean titer; IgA, immunoglobulin A; IgG, immunoglobulin G; RV, rotavirus.

^a^Data represent No. (%) of mother-infant pairs, unless otherwise specified (all *P *> .05).

^b^Note: n = 52 in secretor and n = 20 in nonsecretor group.

^c^Note: n = 51 in secretor and n = 30 in nonsecretor group.

**Table 2.** Seroconversion by Secretor Phenotype

Secretor Phenotype	Total No.	Seroconversion, No. (%)	RR (95% CI)
All infants	246	72 (27)	
Secretor mother	172	40 (23)	
Nonsecretor mother	74	29 (39)	1.69 (1.14–2.50)
Secretor infant	175	51 (29)	
Nonsecretor infant	71	18 (25)	0.82 (.53–1.27)
Secretor infants			
Secretor mother	144	34 (24)	
Nonsecretor mother	31	17 (55)	2.32 (1.50–3.59)
Nonsecretor infants			
Secretor mother	28	6 (21)	
Nonsecretor mother	43	12 (28)	1.30 (.55–3.07)

Abbreviations: CI, confidence interval; RR, relative risk

Controlling for infant secretor status, maternal nonsecretor phenotype remained significantly associated with seroconversion (RR, 2.84; 95% CI, 1.45–5.55). Infant phenotype was not significantly associated with seroconversion after controlling for maternal phenotype (RR, 1.97; 95% CI, .95–4.05). In separate regression analyses, none of the following were significantly associated with infant seroconversion: baseline concentration of infant serum IgA, IgG, breast milk IgA, and duration of peak RV season exposure; in all models, maternal secretor phenotype remained independently associated with seroconversion, and no interactions were observed between variables (data not shown). Limited sample sizes precluded assessment of other known variables or development of a comprehensive model [10]. No association was seen between maternal Lewis positive (a or b) versus Lewis (a and b) negative status and infant seroconversion (28% vs 29%, respectively; RR, 1.01; 95% CI, .79–1.29).

Among 120 mothers with available DNA, 114 (95%) were successfully genotyped, with 6 indeterminate. Twenty mothers (18%) were *se/se* (Supplementary Table 1). Phenotype results concurred with nonsecretor genotype results for all but 1 mother (Supplementary Results). Among 94 *Se/Se* or *Se/se* mothers, 12 (13%) were nonsecretor by phenotype. Maternal *se/se* genotype was also associated with higher rates of seroconversion (50% vs 22%; RR, 2.08; 95% CI, 1.19–3.63). Seroconversion was more frequent in secretor-positive infants of *se/se* mothers (73%) than in secretor-positive infants of *Se/Se or Se/se* mothers (23%; RR, 2.74; 95% CI, 1.63–4.58). Thirty nonsecretor infants had maternal genotype data available, with no difference between seroconversion in those of *se/se* (22%) and *Se/Se* or *Se/se* mothers (19%; RR, 1.39; 95% CI, .31–6.33).

## Discussion

Mechanisms leading to suboptimal oral RV vaccine performance in low- and middle-income countries are not well understood. Using a carefully studied prospective birth cohort of mothers and infants involved in a RV VE trial, we have demonstrated that maternal secretor status significantly affected Rotarix response in a cohort of Bangladeshi infants. Infants of nonsecretor mothers (without expression of 2-fucosylated glycans in their breast milk) were significantly more likely than infants of secretor mothers to seroconvert after immunization with oral RV vaccine. The effect was strongest among secretor-positive infants, with a seroconversion rate of 73% among secretor infants of *se*/*se* mothers. Prior studies have inconsistently correlated infant secretor status with RV vaccine response, but none of those studies controlled for maternal status [4, 11–13]. In the PROVIDE study, year 1 Rotarix VE was reduced in nonsecretors compared with secretors, though this was probably related to low rates of RVD among nonsecretors in the unvaccinated group rather than differences in response to the vaccine [4]. In the current analysis, maternal, but not infant, secretor status remained associated with vaccine response when both were controlled for, implying that maternal contributions may outweigh infant phenotype. Lack of accounting for variations in maternal secretor status might explain conflicting findings regarding infant secretor status on RV vaccine response [11–13].

To our knowledge, this is the first description of the effect maternal secretor status exerts on infant RV vaccine immunogenicity. 2-Fucosylated HMOs have been shown to inhibit the in vitro infectivity of P[8] and P[4] RVs, the most prevalent global RV strains [14]. Our data suggest that similar processes may be occurring in vivo after oral vaccination with Rotarix, a G1P[8] virus. These findings may be attributable to *FUT2*-dependent glycans serving as decoy receptors for vaccine: infants who express these HBGAs on the gut surface without the presence of decoy HMOs delivered by breast milk (ie, secretor infants of nonsecretor mothers) might bind vaccine more efficiently to the gut, leading to improved vaccine response. Alternatively, variations in gut microbiota between infants of secretor-positive and nonsecretor mothers may contribute to differences in vaccine response [15]. Ultimately, promotion of a gut microenvironment permissive for successful vaccination is paramount. Our data suggest that infant HBGA status may play a less essential role in this process, with maternal breast milk status exerting a larger effect around the time of vaccination.

Both child and maternal secretor status are also associated with pathogen-specific wild-type infections. Colston et al [5] found that infant, but not maternal, secretor-positive status was associated with increased risk of asymptomatic wild-type P[8] RV infection through 2 years of age. Maternal contributions may have waned over their longer follow-up period, explaining the contrast with our findings, which was limited to a short period of exclusive breastfeeding. In both that study and the PROVIDE study, no effect of infant secretor status on risk of P[8] RVD was seen [4].

Our findings suggest that Rotarix seroconversion should be increased in populations with a higher overall proportion of nonsecretor mothers, such as Bangladesh (30% vs 20% in white populations), but the opposite is seen in south Asia, where oral vaccines have classically underperformed. Despite the relative benefit of a high rate of maternal nonsecretors, rates of vaccine response remain unacceptably low, underscoring the challenges posed by the myriad additional factors contributing to oral vaccine underperformance. Thus, efforts to improve RV VE may ultimately may require novel vaccination strategies, including next-generation neonatal or parenteral vaccines, because previous interventions to improve current oral vaccine performance within the World Health Organization’s Expanded Program on Immunization schedule have yielded disappointing results. Although our results imply that breast milk components may play a role in inhibiting vaccine response, we do advocate interventions targeting breastfeeding. Withholding breastfeeding for short periods surrounding vaccination is likely insufficient to significantly change the overall gut milieu, and the well-recognized benefits of breastfeeding preclude any consideration of longer-term cessation.

The current study has several limitations. As a substudy of a larger trial, it involves a risk of selection bias. The trial used a delayed Rotarix schedule, potentially limiting generalizability. Seroconversion was defined by samples collected 1 week after the second dose, meaning that results may apply mainly to response to the first dose, owing to insufficient time to reflect plasma responses after dose 2. This study was not powered to detect differences in seroconversion in nonsecretor infants. Because of technical limitations, secretor phenotype analysis was limited to Lewis-positive mothers. Data on infant baseline RV-IgG titers and breast milk RV-IgA concentrations were limited and could mask interactions between important variables potentially associated with seroconversion [10]. RV-IgA is a standard measure of immune response but suboptimally approximates VE [10]. Breast milk HMO concentration varies over time; our samples were limited to a single time point not at the exact time of vaccination. However, confirmation of our phenotype findings using maternal genotype helps confirm their validity.

In conclusion, we demonstrate in a cohort of Bangladeshi infants that maternal, but not infant, secretor status affected oral RV vaccine immunogenicity. Infants of nonsecretor mothers were more likely to seroconvert than infants of secretors. To translate these findings into a revised vaccination strategy for infants, further investigations into specific relationships between breast milk HMOs, mucosal HBGA and viral-glycan binding are necessary. In addition, confirmation of maternal secretor-mediated RV vaccine immunogenicity with clinical vaccine protection is needed. Nevertheless, these data suggest a novel variable that will require consideration for rational next-generation RV vaccine development and in the design and interpretation of future vaccine trials.

## Supplementary Data

Supplementary materials are available at *The Journal of Infectious Diseases* online. Consisting of data provided by the authors to benefit the reader, the posted materials are not copyedited and are the sole responsibility of the authors, so questions or comments should be addressed to the corresponding author.

jiaa101_suppl_Supplementary_Material

## Notes

***Acknowledgments.*** The automated DNA sequencing was performed in the Vermont Integrative Genomics Resource DNA Facility and was supported by University of Vermont Cancer Center, Lake Champlain Cancer Research Organization, and the Larner College of Medicine, University of Vermont.

***Financial support.*** This work was supported by the Bill & Melinda Gates Foundation (grant OPP1127782 to B. D. K.), the Foundation for the Society for Maternal Fetal Medicine (SMFM) (Queenan Fellowship for Global Health to F. B. W.), the International Centre for Diarrhoeal Disease Research, Bangladesh, and the governments of Bangladesh, Canada, Sweden, and the United Kingdom (core/unrestricted support).

***Potential conflicts of interest.*** All authors: No reported conflicts. All authors have submitted the ICMJE Form for Disclosure of Potential Conflicts of Interest. Conflicts that the editors consider relevant to the content of the manuscript have been disclosed.

Presented in part: 10th International Conference on Vaccines for Enteric Diseases, Lausanne, Switzerland, October 16–18, 2019; 68th Annual Meeting of the American Society of Tropical Medicine and Hygiene, National Harbor, Maryland, November 20–24, 2019.

## References

[1] Tate JE, Burton AH, Boschi-Pinto C, et al Global, regional, and national estimates of rotavirus mortality in children <5 years of age, 2000–2013. Clin Infect Dis 2016; 62:S96–105. 27059362 10.1093/cid/civ1013 PMC11979873

[2] Parker EP, Ramani S, Lopman BA, et al Causes of impaired oral vaccine efficacy in developing countries. Future Microbiol 2018; 13:97–118. 29218997 10.2217/fmb-2017-0128 PMC7026772

[3] Huang P, Xia M, Tan M, et al Spike protein VP8* of human rotavirus recognizes histo-blood group antigens in a type-specific manner. J Virol 2012; 86:4833–43. 22345472 10.1128/JVI.05507-11 PMC3347384

[4] Lee B, Dickson DM, deCamp AC, et al Histo–blood group antigen phenotype determines susceptibility to genotype-specific rotavirus infections and impacts measures of rotavirus vaccine efficacy. J Infect Dis 2018; 217:1399–407. 29390150 10.1093/infdis/jiy054 PMC5894073

[5] Colston JM, Francois R, Pisanic N, et al. Effects of child and maternal histo-blood group antigen status on symptomatic and asymptomatic enteric infections in early childhood. J Infect Dis 2019; 220:151–62. 30768135 10.1093/infdis/jiz072 PMC6548901

[6] Colgate ER, Haque R, Dickson DM, et al. Delayed dosing of oral rotavirus vaccine demonstrates decreased risk of rotavirus gastroenteritis associated with serum zinc: a randomized controlled trial. Clin Infect Dis 2016; 63:634–41. 27217217 10.1093/cid/ciw346

[7] Reeck A, Kavanagh O, Estes MK, et al. Serological correlate of protection against norovirus-induced gastroenteritis. J Infect Dis 2010; 202:1212–8. 20815703 10.1086/656364 PMC2945238

[8] Ferrer-Admetlla A, Sikora M, Laayouni H, et al. A natural history of *FUT2* polymorphism in humans. Mol Biol Evol 2009; 26:1993–2003. 19487333 10.1093/molbev/msp108

[9] Silva LM, Carvalho AS, Guillon P, et al. Infection-associated *FUT2* (fucosyltransferase 2) genetic variation and impact on functionality assessed by in vivo studies. Glycoconj J 2010; 27:61–8. 19757028 10.1007/s10719-009-9255-8

[10] Lee B, Carmolli M, Dickson DM, et al. Rotavirus-specific immunoglobulin a responses are impaired and serve as a suboptimal correlate of protection among infants in Bangladesh. Clin Infect Dis 2018; 67:186–92. 29394355 10.1093/cid/ciy076 PMC6030840

[11] Armah GE, Cortese MM, Dennis FE, et al. Rotavirus vaccine take in infants is associated with secretor status. J Infect Dis 2019; 219:746–9. 30357332 10.1093/infdis/jiy573

[12] Pollock L, Bennett A, Jere KC, et al Nonsecretor histo–blood group antigen phenotype is associated with reduced risk of clinical rotavirus vaccine failure in Malawian infants. Clin Infect Dis 2019; 69:1313–9. 30561537 10.1093/cid/ciy1067 PMC6763638

[13] Kazi AM, Cortese MM, Yu Y, et al. Secretor and salivary ABO blood group antigen status predict rotavirus vaccine take in infants. J Infect Dis 2017; 215:786–9. 28329092 10.1093/infdis/jix028

[14] Laucirica DR, Triantis V, Schoemaker R, Estes MK, Ramani S. Milk oligosaccharides inhibit human rotavirus infectivity in MA104 cells. J Nutr 2017; 147: 1709–14. 28637685 10.3945/jn.116.246090 PMC5572490

[15] Lewis ZT, Totten SM, Smilowitz JT, et al. Maternal fucosyltransferase 2 status affects the gut bifidobacterial communities of breastfed infants. Microbiome 2015; 3:13. 25922665 10.1186/s40168-015-0071-z PMC4412032

