# Supplementary material for: Maternal Secretor Status Affects Oral Rotavirus Vaccine Response in Breastfed Infants in Bangladesh
Source: J Infect Dis. 2020 Mar 11;224(7):1147–51. doi: 10.1093/infdis/jiaa101 (PMC8561252; doi:10.1093/infdis/jiaa101)
Supplement: jiaa101_suppl_Supplementary_Material [file jiaa101_suppl_Supplementary_Material.docx]

**SUPPLEMENTARY MATERIALS**

**Methods**

***Breast milk phenotyping*.** Specific antigen detection was performed by EIA using mouse monoclonal antibodies for Lewis a (Gamma-clone, Immucor, USA) and Lewis b (BG-6, clone T218 (Biolegend, USA) [7]. Detection was performed using peroxidase-conjugated goat anti-mouse IgG (Sigma, A2304) followed by development with tetramethylbenzidine (TMB) for optical density measurement.

***FUT2 genotyping.*** Genomic DNA was extracted from available maternal PBMCs using the PicoPure^TM^ kit (Applied Biosystems). The 1,032 bp coding region (exon 2) of *FUT2* was amplified by polymerase chain reaction (PCR) using previously described primers [8] in 25 µL reaction mixtures comprising 3 µL DNA, 2.5 µL of 10X *Taq* polymerase buffer, 50 mM magnesium sulfate, 5 nmol of each primer, 10mM dNTPs and 0.2 µL of *Taq* polymerase (Invitrogen). The conditions used in the reaction were as follows: 10 min of initial denaturation at 96°C, followed by 35 cycles of denaturation at 94°C for 45 s, annealing at 62°C for 45 s and extension at 72°C for 2 min, with a final extension at 72°C for 7 min. Resultant PCR products were visualized using polyacrylamide gel electrophoresis to confirm appropriate band location, and cleaned using ExoSAP-IT (ThermoFisher). Sanger sequencing was performed on cleaned amplicons using forward primer 5′-CCATCTCCCAGCTAACGTGTCC-3′ and reverse primer: 5′-GGGAGGCAGAGAAGGAGAAAAGG-3′ [9] for manual SNP analysis using Geneious Prime 2019.

**Results**

| **Supplemental Table 1. Infant Seroconversion by Maternal Genotype** | | | | | | | | | |  |
| --- | --- | --- | --- | --- | --- | --- | --- | --- | --- | --- |
|  |  |  |  | Non-secretor maternal phenotype | |  | Infant Seroconversion | | |  |
| Maternal Genotype | | Zygosity | No. |  | No. | (%) |  | No. | (%) | RR (95% CI) |
| *Se* |  | 94 |  | 12 | (13) |  | 21 | (22) |  |  |
|  | *Se/Se* | 42 |  | 0 | (0) |  | 9 | (21) |  |  |
|  | *se/Se* | 52 |  | 12 | (23) |  | 12 | (23) |  |  |
| *se* | *se/se* | 20 |  | 19 | (95) |  | 10 | (50) | 1.55 (0.99 - 2.44) |  |
| Abbreviations: RR, relative risk; CI, confidence interval; *Se*, secretor; *se*, non-secretor | | | | | | | | | |  |
|  | | | | | | | | | |  |

***Maternal se/se genotype results.*** Twenty maternal samples were genotyped as s*e/se*, with SNPs in either rs601338G>A or rs200157007C>T. The most prevalent *FUT2* SNP associated with non-secretor status in our population was rs200157007C>T, which accounted for 13/20 *se/se* mothers (11% of all genotyped). Seven (6% of all genotyped) were homozygous for rs601338G>A. The lone *se/se* mother with secretor-positive phenotype was homozygous for rs200157007C>T, with the remaining 12 (92%) phenotypically non-secretor. Since rs200157007C>T encodes a missense mutation rather than a stop mutation, it is possible that some residual enzyme activity could be seen, explaining a variably penetrant secretor phenotype. The twelve *Se/se* or *Se/Se* mothers characterized as non-secretor by phenotype were heterozygous *se/Se* for at least two of the established null alleles, implying that compound heterozygosity may affect breast milk secretor phenotype.
